# Long-Term Consumption of Food-Derived Chlorogenic Acid Protects Mice against Acetaminophen-Induced Hepatotoxicity via Promoting PINK1-Dependent Mitophagy and Inhibiting Apoptosis

**DOI:** 10.3390/toxics10110665

**Published:** 2022-11-05

**Authors:** Bangyan Hu, Jin Li, Daoyin Gong, Yuan Dai, Ping Wang, Lihong Wan, Shijun Xu

**Affiliations:** 1 School of Pharmacy, Chengdu University of Traditional Chinese Medicine, Chengdu 611137, China; 2 State Key Laboratory of Southwestern Chinese Medicine Resources, Chengdu University of Traditional Chinese Medicine, Chengdu 611137, China; 3 Institute of Material Medica Integration and Transformation for Brain Disorders, Chengdu University of Traditional Chinese Medicine, Chengdu 611137, China; 4 School of Health Preservation and Rehabilitation, Chengdu University of Traditional Chinese Medicine, Chengdu 610075, China; 5 Department of Pathology, Hospital of Chengdu University of Traditional Chinese Medicine, Chengdu 610075, China; 6 Department of Pharmacology, West China School of Basic Medical Sciences & Forensic Medicine, Sichuan University, Chengdu 610041, China

**Keywords:** chlorogenic acid, APAP-induced liver injury, apoptosis, PINK1/Parkin pathway, mitophagy

## Abstract

Hepatotoxicity brought on by acetaminophen (APAP) is significantly impacted by mitochondrial dysfunction. Mitophagy, particularly PINK1-mediated mitophagy, maintains the stability of cell function by eliminating damaged mitochondria. One of the most prevalent dietary polyphenols, chlorogenic acid (CGA), has been shown to have hepatoprotective properties. It is yet unknown, nevertheless, whether its defense against hepatocyte apoptosis involves triggering PINK1-mediated mitophagy. In vitro and in vivo models of APAP-induced hepatotoxicity were established to observe CGA’s effect and mechanism in preventing hepatotoxicity in the present study. Serum aminotransferase levels, mouse liver histology, and the survival rate of HepG2 cells and mice were also assessed. The outcomes showed that CGA could reduce the activities of serum enzymes such as alanine transaminase (ALT), aspartate transaminase (AST), and lactate dehydrogenase (LDH), and alleviate liver injury in mice. It could also significantly increase the cell viability of HepG2 cells and the 24-h survival rate of mice. TUNEL labeling and Western blotting were used to identify the hepatocyte apoptosis level. According to data, CGA could significantly reduce liver cell apoptosis in vivo. Additionally, Tom20 and LC3II colocalization in mitochondria may be facilitated by CGA. CGA considerably increased the levels of genes and proteins associated with mitophagy (PINK1, Parkin, LC3II/LC3I), while considerably decreasing the levels of p62 and Tom20, suggesting that it might activate PINK1/Parkin-mediated mitophagy in APAP-induced liver damage. Additionally, the protection of CGA was reduced when PINK1 was knocked down by siPINK1 in HepG2 cells, and it did not upregulate mitophagy-related proteins (PINK1, Parkin, LC3II/LC3I). In conclusion, our findings revealed that long-term consumption of food-derived CGA could prevent APAP hepatotoxicity via increasing PINK1-dependent mitophagy and inhibiting hepatocyte apoptosis.

## 1. Introduction

**Figure 1:**
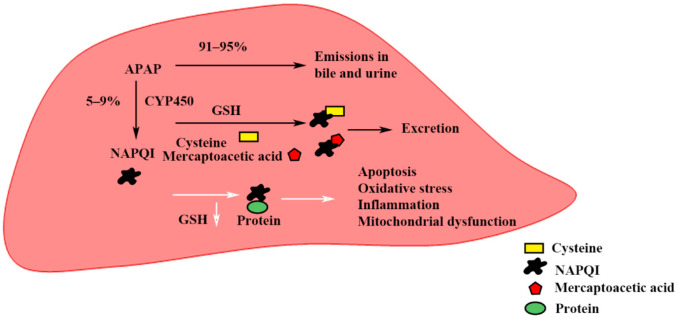
Schematic diagram of APAP-induced hepatotoxicity. NOPQI is metabolized in combination with cysteine and mercaptoacetic acid when GSH is abundant. With the depletion of GSH, the accumulated NAPQI binds to some large proteins to induce mitochondrial damage, oxidative stress, an inflammatory response, and apoptosis.

Acetaminophen (APAP) overdoses are one of the most frequent forms of drug-induced liver injury (DILI), which poses a serious threat to public health. Since 1955, APAP has been a widely used antipyretic and analgesic medication. As the liver is one of the most sensitive organs to APAP, even if it is normally safe at a therapeutic dose (4 g per 24 h), APAP could result in serious liver damage through hepatotoxicity with a single excess dose reaching 15 to 25 g [1,2]. In the United States, APAP overdoses account for 50% of all occurrences of acute liver failure, and they are the second most common reason for liver transplants in people worldwide [3,4,5]. The hallmark of APAP hepatotoxicity is the production of mitochondrial superoxide and peroxynitrite. It is widely known that APAP hepatotoxicity results from the overproduction of the poisonous metabolite N-acetyl-p-benzoquinone imine (NAPQI), even though Cytochrome P450 only converts a very small amount of APAP (5–9%) into NAPQI at therapeutic doses [6]. Apoptosis, glutathione (GSH) depletion, oxidative stress, sterile inflammation, mitochondrial dysfunction, and excessive NAPQI buildup are all caused by the metabolism of overdose APAP by P450, which in turn causes hepatotoxicity and hepatic necrosis [7,8,9,10] (Figure 1).

The major target of APAP hepatotoxicity is the mitochondria, which are highly dynamic organelles that regulate cell redox homeostasis, innate immunity, and apoptosis [11,12]. These organelles also serve as the origin of inflammatory signals and oxidative stress responses in liver cells [13]. The primary cause of liver apoptosis and death in APAP hepatotoxicity is mitochondrial dysfunction [14]. Emerging data suggest that mitophagy is essential to the physiology and pathology of the liver [15]. Mitophagy can maintain its function, metabolic stability, and reactive oxygen species (ROS) equilibrium while selectively destroying mitochondria damaged by ROS [16]. Typically, autophagosome production, mitochondrial fission, and fusion with lysosomes occur during mitophagy [17]. The PINK1/Parkin pathway, which is activated by the phosphatase and tenson homolog (PTEN) gene, controls these functions. In brief, in answer to various stimuli, PINK1 locates damaged mitochondria from the inner membrane on the outside mitochondrial membrane, and then, by recruiting Parkin, removes the damaged mitochondria [18]. Importantly, Pink1/Parkin suppression increases APAP hepatotoxicity by impairing hepatic mitophagy, suggesting that Pink1/Parkin-mediated mitophagy may be essential for reducing APAP toxicity [19,20]. Therefore, it is crucial to precisely regulate the Pink1/Parkin pathway to prevent APAP hepatotoxicity. Intriguingly, a recent study discovered that knockdown of PINK1 dramatically decreased mitophagy in a cell model of cadmium-induced mitochondrial dysfunction and exacerbated liver parenchymal cell damage [21]. In hypoxia/reoxygenation-induced L02 cells, cell apoptosis was increased after PINK1 was knocked down by siRNA, suggesting that PINK1-mediated mitophagy plays a role in controlling apoptosis [22]. However, the effectiveness of mitophagy-mediated apoptosis in reducing APAP hepatotoxicity is still mainly unknown.

N-acetylcysteine (NAC) is currently thought to be the only antidote for APAP due to its potent antioxidant effects by boosting the content of endogenous GSH [23,24]. Due to NAC’s drawbacks, such as a narrow therapeutic window and limited effectiveness, it is vital and necessary to develop early prevention and intervention measures that can prevent APAP hepatotoxicity, particularly natural bioactive substances [25,26]. Ammonium glycyrrhizinate (AG), also known as glycyrrhizin or glycyrrhetinic acid, is an inhibitor of high mobility group box 1 (HMGB1) protein with antioxidant activity and has been reported to protect against APAP-induced liver toxicity through tumor necrosis factor α (TNF-α)-mediated apoptosis and the fatty acid metabolic pathway [27,28,29,30,31]. Thus, it was used as a positive drug in this study. Several studies have shown that phenolic phytochemicals in dietary foods, spices, and herbs with antioxidant activity can prevent the liver toxicity caused by APAP [32,33]. Chlorogenic acid (CGA) is a potent antioxidant with a purportedly high safety profile that is a naturally occurring dietary polyphenolic component produced from coffee, apple, blueberries, tea, and several natural remedies such as Lonicerae flos. According to reports, CGA levels in coffee, sunflower seeds, and Lonicerae flos range from 2.0% to 8.00%, 1.50 to 3.00%, and 1.00 to 5.90%, respectively [34]. The protective effect of CGA on APAP hepatotoxicity has been demonstrated by increasing data. This impact incorporates many mechanisms, including anti-oxidation, anti-inflammatory, and anti-apoptosis [9,35,36,37]. CGA’s unique strategies against APAP hepatotoxicity include blocking CYP2E1 and CYP1A2 enzymatic characteristics, attenuating liver mitochondrial injury and lowering mitochondrial HSP60 production, activating the Nrf2 anti-oxidative signaling system, and inhibiting the MAPK, TLR3/4, and NF kappa B signaling pathways [9,38,39,40,41,42,43,44]. Cellular homeostasis and the response of the mitochondria to stress are significantly influenced by mitophagy. The fundamental mechanisms of oxidative stress, inflammation, and apoptosis are mitophagy abnormalities. However, it is unclear whether long-term consumption of food-derived CGA can trigger mitophagy dependent on PINK1, which suppresses liver cell death to reduce APAP hepatotoxicity. Determining the underlying mechanism and evaluating the hepatoprotective impact of CGA in overdose APAP-induced mice are the goals of the current investigation.

## 2. Materials and Methods

### 2.1. Chemicals and Reagents

CGA (purify ≥ 98%) was purchased from Herbest Co., Ltd. (Baoji, China). APAP was obtained from Shanghai Johnson Pharmaceutical Co., Ltd. (Shanghai, China). AG (purify ≥ 98%) was provided by Abphyto Co., Ltd. (Chengdu, China). Commercial assay kits for alanine transaminase (ALT), aspartate transaminase (AST), and lactate dehydrogenase (LDH) were supplied by Medical System Co., Ltd. (Ningbo, China). A Cell Total RNA Isolation Kit was acquired from Foregene Co., Ltd. (Chengdu, China). Anti-Tom20 was provided by Santa Cruz Biotechnology, Inc. Anti-SQSTM1/p62 and anti-Parkin were offered by Cell Signaling Technology (Danvers, MA, USA). Anti-LC3II, anti-Bcl2 and anti-Bax were provided by Proteintech Group, Inc. (Wuhan, China). Anti-PINK1 was purchased from Abcam (Cambridge, UK). Anti-glyceraldehyde phosphate dehydrogenase (GAPDH), Alexa Fluor^®^ 488 AffiniPure, Goat Anti-Rabbit IgG(H+L) Alexa Fluor^®^, Alexa Fluor^®^ Cy3 AffiniPure Goat Anti-Mouse IgG(H+L) Alexa Fluor^®^, Goat Anti-Mouse or anti-Rabbit IgG (H+L) Secondary Antibody HRP, and the terminal deoxynucleotidyl transferase-mediated dUTP-biotin nick end labeling (TUNEL) kit were produced by Servicebio (Wuhan, China). The BCA protein assay kit was acquired from Multi Sciences (Hangzhou China).

### 2.2. Animals

**Figure 2:**
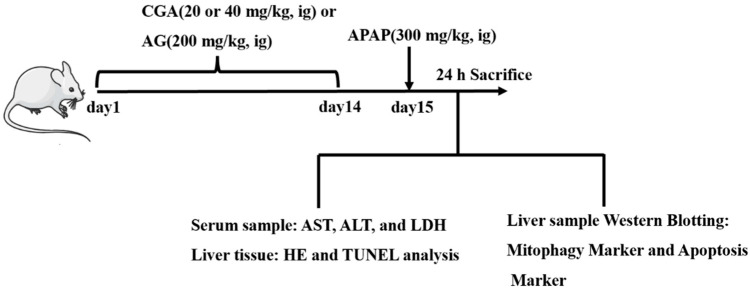
A schematic diagram of the treatment schedule.

One hundred male Kunming mice (weighing 20 ± 2 g, 4–6 weeks age) were obtained from Byrness Weil Biotech Co., Ltd. (No.: 0003181, Chengdu, China). After all mice were acclimated for 3 days in a constant temperature and humidity room (24 °C ± 1 °C, 50% ± 10% humidity) with a standard diet, water, and a 12-h light/dark cycle (lights on at 8:00 am and lights off at 8:00 pm), animals were randomly separated into five groups (*n* = 20): the control group (Ctrl), APAP (300 mg/kg), the APAP (300 mg/kg) + AG group (200 mg/kg), and the APAP (300 mg/kg) + CGA group (20 mg/kg or 40 mg/kg). Mice in the intervention group were respectively pre-administered (i.g.) with CGA (20 mg/kg or 40 mg/kg) or AG (200 mg/kg) for 14 consecutive days. Simultaneously, the Ctrl and APAP groups were congruously orally administrated (i.g.) with the same volume of 0.9% saline. On day 15, mice were orally administered (i.g.) a single dose of APAP (300 mg/kg) to induce acute hepatotoxicity injury, except the Ctrl group, which was orally administered (i.g.) 0.9% saline. The dose for APAP-induced liver injury was sourced from the reported literature [45,46]. After APAP (300 mg/kg) treatment, the death period of mice was recorded within 24 h. All mice were anesthetized with pentobarbital (50 mg/kg, i.p., dissolved in sterilized normal saline) on day 16, blood was collected by cardiac puncture, and serum was obtained after centrifugation at 3500 rpm for 10 min at room temperature. Then, all animals were euthanized and liver tissues were collected. A schematic diagram of the treatment schedule is shown in Figure 2. Animal care and experimental protocols were approved by the Ethics Committee for Animal Experiments of the Institute of Material Medica Integration and Transformation for Brain Disorders, Chengdu University of Traditional Chinese Medicine (Permit No.: IBD2019008, 1 September 2019, Chengdu, China).

### 2.3. Cell Culture

HepG2 cell lines were purchased from the Cell Bank of the Chinese Academy of Science (Shanghai, China). HepG2 cells were cultured in DMEM with 10% fetal bovine serum and 1% penicillin/streptomycin (Gibco) at 37 °C with 5% CO_2_. The cells were divided into Ctrl, APAP, APAP + CGA, APAP + CGA + siNC, APAP + CGA + siPINK1. Then, 1 × 10^4^ cells cultured in 96-well plates were pre-incubated with CGA at different concentrations for 15 min after adherence, and then incubated with APAP for an additional 24 h. Cell viability was measured by MTT assay according to the manufacturer’s instructions.

### 2.4. Biochemical Analysis for Serum

Since alanine aminotransferase (ALT), aspartate aminotransferase (AST), and alkaline phosphatase (ALP) are the classic diagnostic markers of liver injury [47], the serum enzymatic activities of ALT, AST, and LDH were measured with a HITACHI 7180 automatic biochemistry analyzer (Hitachi, Japan).

### 2.5. HE Staining and TUNEL Assay

Fresh liver tissues were immediately fixed in 4% paraformaldehyde for 24 h, embedded in paraffin, and then cut into 4-μm-thick sections. The tissues were stained with hematoxylin and eosin (HE) for histological examination under light microscopy. The histological scores (including inflammation and necrosis) indicating the degree of liver injury were determined according to Suzike’s standard in a blinded manner [48]. The scoring criteria are as follows: the score scale of inflammation ranged from 0 to 4, indicating none, slight, mild, moderate, and severe, respectively. The score scale of necrosis ranged from 0 to 4, indicating no necrosis area, a single cell, <30%, 31–60%, >60%. Simultaneously, terminal deoxynucleotidyl transferase-mediated dUTP-biotin nick end labeling (TUNEL) was used to measure hepatic apoptosis according to the instructions of the manufacturer of the TUNEL apoptotic detection kit. Images were captured by fluorescence microscopy (Leica Microsystems, Wetzlar, Germany), and we counted the positive cells.

### 2.6. PINK1 siRNA Transfection

PINK1-siRNA (Hanheng Biotechnology, Shanghai, China) was used to knock down PINK1 in HepG2 cells, according to the manufacturer’s instructions. In addition, HepG2 cells transfected with non-silencing scrambled siRNA (siNC, Hanheng Biotechnology. Shanghai, China) were used as the Ctrl. The respective siRNA senses and antisense sequences for PINK1 and Ctrl siRNA were as follows: siPINK1, 5′-CGCUGUUCCUCGUUAUGAATT-3′ and 5′-UUCAUAACGAGGAACAGCGTT-3′, and siNC, 5′-UUCUCCGAACGUGUCACGUdTdT-3′ and 5′-ACGUGACACGUUCGGAGAA dTdT-3′. After the cells were successfully transfected, the cells were treated according to the method shown in Section 2.7, Section 2.8 and then subjected to RT-qPCR and Western blot analysis.

### 2.7. RT-qPCR Analysis

Total RNA was extracted from liver samples using Trizol reagent. cDNA was synthesized with the SuperScript^®^ IV First-Strand Synthesis System (Bio-Rad, Singapore). RT-qPCR was performed with the SYBR-Green PCR kit on the Applied Biosystems StepOnePlus system. β-actin was used as the invariant Ctrl. The target gene expression levels were obtained using the 2^−ΔΔCt^ method. The sequences of primers used in this study were as follows: PINK1: Forward 5′-AGACTCCCAGTTCTCGCCT-3′, Reverse 3′- AGGGACAGCCATCTGAGTCC-5′; Parkin: Forward5′-AGCCAGAGGTCCAGCAGTT-3′, Reverse 3′-CTGGCACTCACCACTCATCC-5′; p62: Forward 5′-AGATAGCCTTGGAG TCGGTG-3′, Reverse 3′-CCGGGGATCAGCCTC TGTAG-5′; LC3-II: Forward 5′-ACCCTAACCCCATAGGAGCC-3′, Reverse 3′-TGCAAGCGCCGTCTGATTA-5′; β-actin: Forward 5′-GCTCCGGCATGTGCAAAG-3′, Reverse 3′-TTCCCACCATCACACCC TGG-5′.

### 2.8. Western Blotting Analysis

The total proteins of the liver were extracted using RIPA lysis buffer containing protease and phosphatase inhibitors (100:1:1). Protein concentrations were determined using the BCA protein assay kit. Equal amounts of total protein (30 μg) were separated by 8–15% SDS-PAGE gels before transfer to PVDF membranes. Then, the membranes were blocked with 5% fat-free milk at room temperature for 90 min and incubated with primary antibodies at 4 °C overnight, including anti-GAPDH (1:5000), anti-Parkin (1:1000), anti-PINK1 (1:1000), anti-p62 (1:1000), anti-LC3 (1:1000), anti-Bcl-2 (1:1000), and anti-Bax (1:1000). After washing with TBST, the membranes were incubated with secondary goat anti-rabbit IgG (1:5000) or anti-mouse IgG (1:5000) at room temperature for 90 min, and were conjugated with horseradish peroxidase. Then, proteins were detected with chemiluminescence reagent. The relative protein levels were normalized to the GAPDH level.

### 2.9. Immunofluorescence Analysis

The liver tissue was cut into 4-μm-thick sections, followed by deparaffinization with xylene and gradient ethanol, antigen retrieval with trisodium citrate dihydrate for 30 min, and blocking with 1% BSA at room temperature for 30 min. The sections were incubated with primary anti-Tom20 (1:100) and anti-LCII (1:300) at 4 °C overnight, and then with secondary antibodies (1:500) in the dark for 1 h. Hoechst 33258 was used to counterstain the nucleus. Lastly, the sections were mounted with anti-fluorescence quenching sealer and observed via confocal microscopy (Olympus, Japan).

### 2.10. Statistical Analysis

Statistical analysis was performed with one-way analysis of variance (ANOVA), followed by Bonferroni’s post-tests. The values were expressed as the mean ± standard error of the mean (SEM). The survival rate was analyzed via the log-rank test. Values of *p <* 0.05 were considered to be statistically significant. The R language was applied for the statistical analysis and graph work.

## 3. Results

### 3.1. CGA Alleviated Hepatotoxicity in APAP-Induced Mice

**Figure 3:**
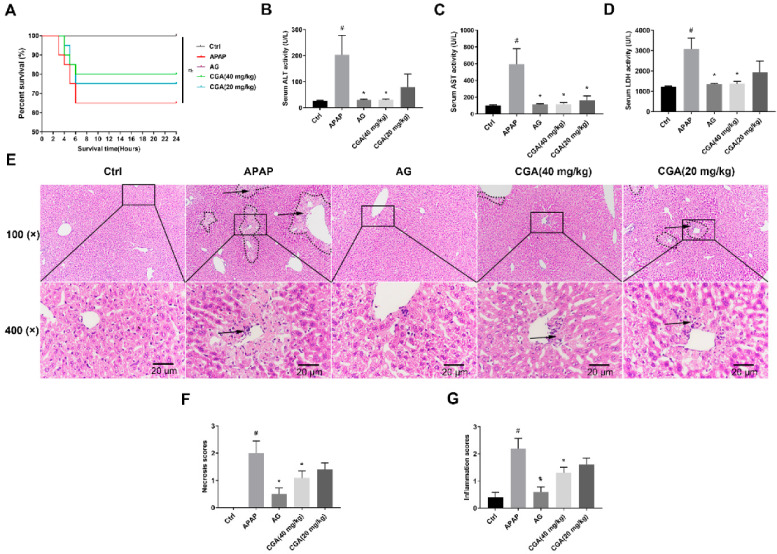
CGA alleviated hepatotoxicity in APAP-induced mice. (**A**) 24 h survival rate of APAP-induced liver injury mice (log-rank test, *n* = 20). (**B**–**D**) Serum levels of ALT, AST, and LDH (*n* = 5). (**E**) Microscopic pictures of the H&E-stained hepatic sections (*n* = 5, scale bar = 20 μm); the arrows indicate leukocyte infiltration and the dotted line represents the necrotic area. (**F**) Histological necrosis scores (*n* = 5). (**G**) Inflammation scores (*n* = 5). Data are displayed as mean ± S.E.M. ^#^ *p* < 0.05 vs. Ctrl group; * *p* < 0.05 vs. APAP group.

To evaluate the preventive effect of CGA in APAP-induced liver injury, the 24 h survival rates of mice, the activities of serum enzymes (AST, ALT, LDH), and liver injury were assessed. As shown in Figure 3A, APAP administration resulted in an obvious decline in survival rates to 65% within 6 h, which remained at this level for up to 24 h. Pretreatment with CGA (40 mg/kg) or AG increased survival rates compared with APAP alone. The activities of serum ALT, AST, and LDH in APAP hepatotoxicity mice were significantly increased compared with the Ctrl group, while markedly decreased in the AG and CGA pretreatment (40 mg/kg) group (*p <* 0.05, Figure 3B–D). As shown in Figure 3E, APAP administration significantly induced severe hepatocellular injury compared with the Ctrl group, such as the loss of hepatocyte architecture, vacuolization of hepatocytes, massive necrosis, and mononuclear cell infiltration in the portal area. Conversely, pretreatment with CGA or AG significantly ameliorated the hepatocellular injury around the portal area. The results of the necrosis score and inflammation score showed high conformity with HE staining (*p <* 0.05, Figure 3F,G). These results suggested that pretreatment with CGA could prophylaxis APAP hepatotoxicity in mice.

### 3.2. CGA Suppressed Liver Cell Apoptosis in APAP Hepatotoxicity Mice

**Figure 4:**
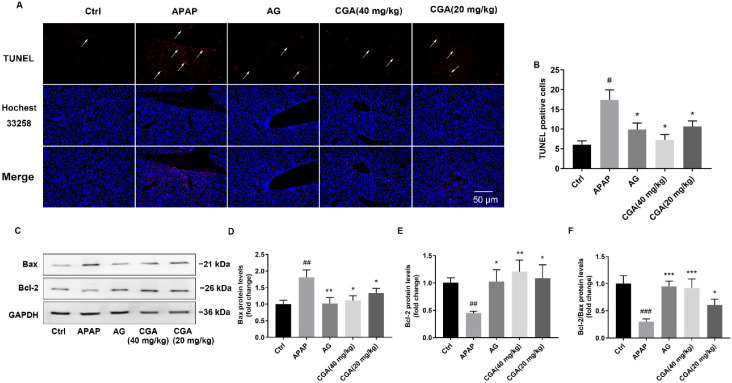
CGA suppressed liver cell apoptosis in APAP hepatotoxicity mice. (**A**) Representative microscopic images of liver cell apoptosis (TUNEL assay, 200 (×), scale bar = 50 μm); the arrows indicate apoptotic cells. (**B**) TUNEL-positive stained cells (*n* = 4). (**C**) Representative Western blotting images of Bcl-2 and Bax in liver tissue. GAPDH was used as an internal standard. (**D**–**F**) Quantitative analysis of Bcl-2, Bax, and the ratio of Bcl-2/Bax in the liver in different groups by Bio-Rad Quantity One v4.62 software. ^#^ *p* < 0.05, ^##^ *p* < 0.01, ^###^ *p* < 0.001 vs. Ctrl group; * *p* < 0.05, ** *p* < 0.01, *** *p* < 0.001 vs. APAP group.

Since hepatocyte apoptosis is the key feature of APAP hepatotoxicity, TUNEL staining and Western blotting were used to evaluate the CGA against apoptosis. As shown in Figure 4A,B, there were few apoptotic cells in the normal liver tissue. However, significantly increased numbers of apoptotic cells could be observed in the APAP group, which was significantly reduced by pretreatment with CGA or AG (*p <* 0.05). To further demonstrate the effect of CGA on APAP-induced apoptosis, the expression of apoptosis-associated proteins (Bax and Bcl-2) was measured by Western blotting. Our data showed that APAP mice presented remarkably elevated expression of Bax and decreased expression of Bcl-2 (*p <* 0.01, Figure 4C–E) and a reduced Bcl-2/Bax rate (*p <* 0.001, Figure 4F) compared to the Ctrl group, which were significantly reversed after treatment with CGA (40 mg/kg) or AG (*p <* 0.05, *p <* 0.01, or *p <* 0.001, Figure 4C–F). These results indicated that CGA attenuated APAP-induced apoptosis of hepatocytes by regulating Bcl-2 family protein levels in mice.

### 3.3. CGA Triggered PINK1-Dependent Mitophagy in APAP Hepatotoxicity Mice

**Figure 5:**
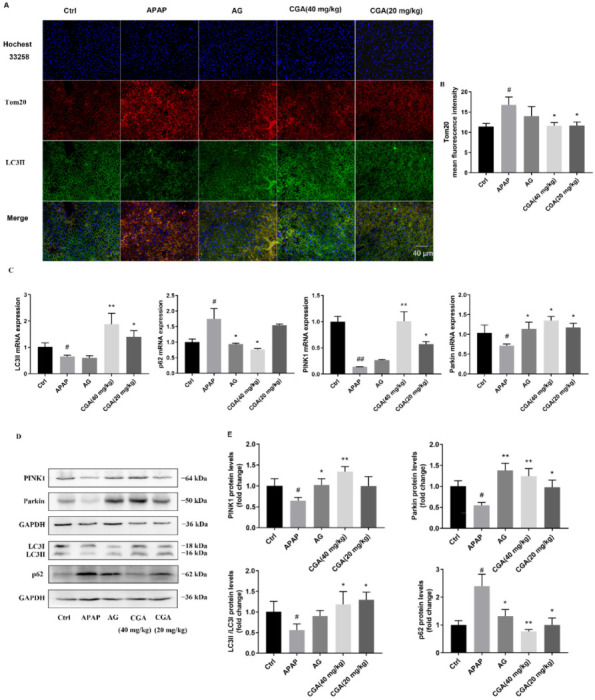
CGA triggered PINK1-dependent mitophagy in APAP hepatotoxicity mice. (**A**) Representative immunofluorescence images of LC3II and Tom20 (*n* = 4, scale bar = 40 μm). (**B**) Quantitative analysis of Tom20 mean fluorescence intensity by Image J. (**C**) The expression of PINK1 mRNA, Parkin mRNA, LC3II mRNA, and p62 mRNA (*n* = 3). (**D**) Representative Western blotting images of PINK1, Parkin, LC3I, LC3II, and p62 in liver. GAPDH was used as an internal standard. (**E**) Quantitative analysis of PINK1, Parkin, LC3II/LC3I, and p62 in liver in different groups by Bio-Rad Quantity One (*n* = 3). ^#^ *p* < 0.05, ^##^ *p* < 0.01 vs. Ctrl group; * *p* < 0.05, ** *p* < 0.01 vs. APAP group.

PINK1-dependent mitophagy is closely related to apoptosis. In order to clarify whether CGA, acting against liver apoptosis, is involved in activating mitophagy through the PINK1/Parkin pathway, mitophagy was evaluated by immunofluorescence colocalization, RT-qPCR, and Western blotting. Since the expression decrease of Tom20 (a specific mitochondria marker) is often considered to be due to the reduction in mitochondria damage and the activation of autophagy [49], the colocalization of Tom20 and LC3II (autophagy marker) was firstly measured by immunofluorescence analysis. The results showed that pretreatment with CGA significantly promoted the colocalization of Tom20 and LC3II in the membranes of the mitochondria to exert a mitophagy-enhancing effect compared with APAP mice (Figure 5A,B, *p <* 0.05). Moreover, the expression of LC3II mRNA and protein was dramatically increased, while p62 showed the opposite compared with APAP mice; the ratio levels of LC3II/I were significantly decreased in APAP mice, while they were remarkably increased in CGA mice (*p <* 0.05 or *p <* 0.01, Figure 5C–E). These results indicated that CGA promoted autophagy flux in APAP-induced liver injury mice. Furthermore, PINK1-dependent mitophagy was recently recognized as a novel target for the treatment of alcoholic liver disease and APAP hepatotoxicity [20,50]. The gene and protein expression of PINK1 and Parkin were significantly downregulated in the APAP group compared with the Ctrl group, while being markedly upregulated in the group that received pretreatment with CGA (*p <* 0.05 or *p <* 0.01, Figure 5C–E). These results confirmed that the PINK1-depended mitophagy deficit is the underlying mechanism of overdose APAP hepatotoxicity, and CGA protects against APAP hepatotoxicity by activating PINK1-depended mitophagy.

### 3.4. siPINK1 Reversed the Protective Effect of CGA

**Figure 6:**
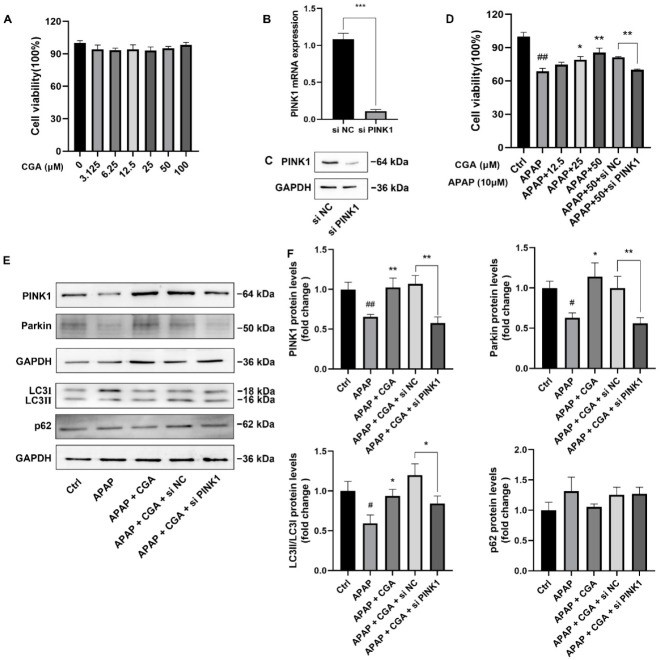
siPINK1 reversed the protective effect of CGA in APAP-induced HepG2 cells. (**A**) Cell viability: HepG2 cells were treated with different concentrations of CGA for 24 h, and cell viability was measured by MTT assay. (**B**,**C**) The expression of PINK1 mRNA and protein in siPINK1 HepG2 cells. (**D**) Cell viability: Normal HepG2 cells, as well as HepG2 cells transfected with siPINK1 or siNC, were exposed to APAP conditions with or without CGA for 24 h, and cell viability was measured by MTT assay. (**E**) Representative Western blotting images of PINK1, Parkin, LC3, and p62 in HepG2 cells. GAPDH was used as an internal standard. (**F**) Quantitative analysis of PINK1, Parkin, LC3II/LC3I, and p62 in HepG2 in different groups by Bio-Rad Quantity One (*n* = 3). ^#^ *p* < 0.05, ^##^ *p* < 0.01 vs. Ctrl group; * *p* < 0.05, ** *p* < 0.01 vs. APAP group.

To further confirm that the protection against APAP hepatotoxicity is dependent on the promotion of PINK1-dependent mitophagy, siPINK1 HepG2 cells (*p* < 0.001, Figure 6B,C) were established. Our data showed that CGA had no significant effect on the viability of HepG2 cells at 0–100 μM (Figure 6A), while cell viability was significantly increased by CGA (25–50 μM) in APAP (10 mM)-induced HepG2 cells (*p <* 0.05 or *p <* 0.01, Figure 6D), suggesting that CGA could protect against APAP cytotoxicity in vitro. Moreover, the cytoprotective effect of CGA was reversed when PINK1 was knocked down by siRNA (*p <* 0.01, Figure 6D). As expected, the expression of mitophagy-related proteins (PINK1, Parkin, LC3II/I ratio) was also reversed, with the exception of p62 (*p* < 0.05 or *p <* 0.01, Figure 6E, F). These results, gained from siPINK1 HepG2 cells, further demonstrated that CGA protected against APAP hepatotoxicity in a PINK1-dependent manner, triggering mitophagy.

## 4. Discussion

**Figure 7:**
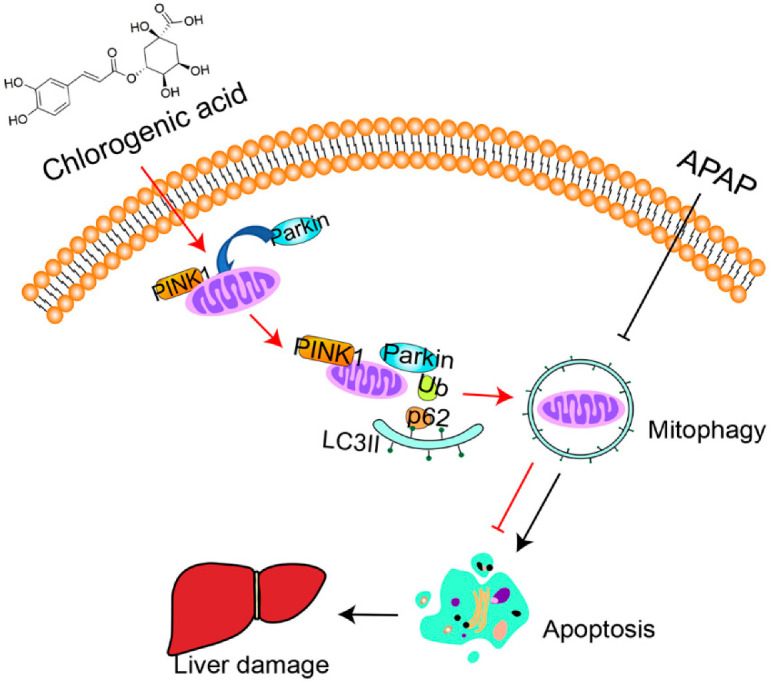
Graphical summary of the results. CGA protects against acetaminophen-induced hepatotoxicity in mice through activating PINK1-dependent mitophagy to inhibit apoptosis.

The current investigation proved that insufficient PINK1-dependent mitophagy was one of the primary mechanisms of APAP-induced hepatotoxicity and demonstrated the preventive efficacy of CGA in APAP-induced liver injury by activating PINK1-dependent mitophagy and inhibiting apoptosis (As shown in Figure 7).

As a non-opioid analgesic, APAP frequently causes toxicity, most notably hepatotoxicity. Due to its accessibility and false perceptions of its safety, hepatotoxicity induced by APAP overdose is a highly regular occurrence throughout the world. The only antidote to APAP overdose currently approved by the FDA is NAC. However, it has a limited therapeutic window and is only effective when taken within 8 h of consuming APAP [46,51]. This deficiency emphasizes the critical need for novel, late-acting medicines. In this context, natural active ingredients have already received a lot of attention in recent decades. Some natural components, such as ginsenoside Rk3, rosmarinic acid, isorhamnetin, and Emodin, have been found to have hepatoprotective potential in preventing APAP-induced liver injury [52,53,54,55]. AG has been shown to prevent liver injury via binding to and inhibiting HMGB1, decreasing TNF-mediated apoptosis, and inhibiting the fatty acid metabolic pathway [27,28,29,30,31]. In patients with drug-induced liver injury, compound glycyrrhizin’s injection, an AG preparation, has a positive hepatoprotective effect by lowering the levels of ALT and AST [56,57]. AG (200 mg/kg) was chosen as a positive medication in this study based on the available literature [31]. Our data showed that AG preadministration significantly reduced APAP-induced hepatotoxicity by decreasing the activities of serum ALT, AST, and LDH, as well as hepatocyte apoptosis.

Plant polyphenols from food (such as CGA) have been shown to protect against and even prevent liver damage thanks to their benefits as natural antioxidants and their low toxicity. It has been demonstrated that the phenylpropanoid molecule CGA, which has significant antioxidant capacity, can prevent liver damage brought about by a variety of medications, including trimethylamine-N-oxide production, thioacetamide, carbon tetrachloride, methotrexate, and APAP [38,58,59,60]. In this experiment, the dose of CGA in mice was 20–40 mg/kg, which equates to approximately 133–266 mg/day in the human body. Our data showed that CGA (20 to 40 mg/kg) could protect mice against acetaminophen-induced hepatotoxicity. Since CGA is a dietary polyphenolic component that is present in many foods, such as coffee (70–350 mg/ cup), apples (0.615–1.181 mg/g), and blueberries (0.85 mg/g) [61,62,63], it is relatively easy to obtain sufficient amounts of CGA from food daily. According to our data, CGA pretreatment dramatically increased survival rates, and liver function was shown to be enhanced by preventing the increase in serum ALT, AST, and LDH that an overdose of APAP causes. In addition, APAP-induced HepG2 cells’ cell viability was improved by CGA. Hepatocyte apoptosis and necrosis coexistence have been well-documented as being crucial to APAP overdose-induced hepatotoxicity since it results in ongoing liver damage [64,65]. Apoptosis is typically started by mitochondrial malfunction and is controlled by Bax and Bcl-2 [66]. Results from the TUNEL and Western blotting analyses in the current study demonstrated that CGA dramatically reduced the number of apoptotic cells and reversed the high Bax level and lowered the Bcl-2 level, showing that CGA had anti-apoptosis effects on the hepatotoxicity of APAP. These findings imply that the long-term consumption of food with high content of CGA results in a lower risk of APAP hepatotoxicity.

Autophagy modulates hepatic apoptosis to determine cell fate via complex cross-talk signals. Hepatocyte apoptosis and autophagy may overlap in liver damage, according to recent findings [67]. Limiting autophagy can reduce the hepatotoxicity produced by APAP, but liver damage from APAP treatment worsens after autophagy is stopped by p62 KO 24 h later [68,69]. Inadequate ATP synthesis is caused by mitochondrial malfunction, which also raises mitochondrial ROS, releases an excessive amount of cytochrome c (Cyt-c), upregulates Bax, caspase-3, and caspase-9, and starts the death cascade [70]. As expected, APAP overdose greatly hindered shifts of the LC3 protein from cytoplasmic to autophagosome form (LC3II) and markedly elevated p62 and Tom20 levels, whereas CGA therapy reversed the adverse effects of APAP overdose and significantly boosted LC3II expression on mitochondria. These results suggested that CGA protected against the cytotoxicity of APAP by inhibiting liver cell apoptosis while increasing hepatic autophagy.

APAP overdose frequently causes hepatotoxic insults by damaging mitochondria and increasing ROS. Traditionally, ATP generation and the creation or scavenging of free radicals occur in mitochondria. By eliminating ROS-damaged mitochondria, mitophagy maintains the equilibrium of mitochondrial function and ROS [16], indicating that mitochondria are a major target for APAP hepatotoxicity. According to recent research, mitophagy, particularly PINK1-mediated mitophagy, is crucial in clearing damaged mitochondria during APAP hepatotoxicity [71]. The PINK1/Parkin pathway, a well-known signaling pathway protein that controls mitophagy, can remove harmed or dysfunctional mitochondria [72,73]. Our results demonstrated that APAP decreased the expression of PINK1 and Parkin, as well as LC3II and LC3I, in vivo and in vitro, and increased the colocalization of LC3II and Tom20 on mitochondria, indicating that a PINK1/Parkin-mediated mitophagy deficit participates in and exacerbates APAP hepatotoxicity. Parkin is recruited by PINK1 to the mitochondrial membrane, starting the PINK1/Parkin-mediated mitophagy process [74]. Tom20 is a particular mitochondrial marker, and its expression is frequently linked to reduced mitochondrial damage and increased autophagy. Surprisingly, our findings revealed that CGA dramatically stimulated, in APAP-induced mice, increased levels of PINK1, Parkin, and LC3II/LC3I; reduced the levels of p62; and decreased the colocalization of LC3II and Tom20 on mitochondria, which were signs of mitophagy. The action of CGA in APAP-induced HepG2 cells was largely consistent with mouse studies. Additionally, the protective effect of PINK1 and Parkin increases due to CGA was reversed once PINK1 was knocked down by siPINK1 in vitro. These results suggested that the long-term consumption of food-derived CGA could prevent APAP hepatotoxicity by inducing mitophagy in a way that depends on PINK1. The present study has certain limitations, such as the question of whether CGA can lessen APAP hepatotoxicity when administered therapeutically and in age-matched mice for a certain number of weeks. This will be the direction of our future research.

## 5. Conclusions

A crucial mechanism by which APAP causes hepatotoxicity, as demonstrated by the current work, is via increasing apoptosis by inhibiting PINK1-dependent mitophagy. CGA could prevent APAP hepatotoxicity by activating PINK1-dependent mitophagy, inhibiting apoptosis, and decreasing serum aminotransferase activity. Our findings imply that the long-term consumption of foods that have high content of CGA may prevent APAP hepatotoxicity.

## Data Availability

The data presented in the present study are available on request from the first author or corresponding author. All data are not publicly available due to privacy.
